# Comparison of viral communities in the blood, feces and various tissues of wild brown rats (*Rattus norvegicus*)

**DOI:** 10.1016/j.heliyon.2023.e17222

**Published:** 2023-06-13

**Authors:** Zi Zhuang, Lingling Qian, Juan Lu, Xiaodan Zhang, Asif Mahmood, Lei Cui, Huiying Wang, Xiaochun Wang, Shixing Yang, Likai Ji, Tongling Shan, Quan Shen, Wen Zhang

**Affiliations:** a Department of Laboratory Medicine, School of Medicine, Jiangsu University, Zhenjiang, 212013, China; b Department of Clinical Laboratory, Zhenjiang Center for Disease Prevention and Control, Zhenjiang, 212002, China; c Institute of Animal Husbandry and Veterinary Science, Shanghai Academy of Agricultural Sciences, Shanghai, 200062, China; d Department of Swine Infectious Disease, Shanghai Veterinary Research Institute, Chinese Academy of Agricultural Sciences, Shanghai, 200241, China

**Keywords:** Wild *Rattus norvegicus*, Viral metagenomics, Virome, Zhenjiang

## Abstract

Viral diseases caused by new outbreaks of viral infections pose a serious threat to human health. Wild brown rats (*Rattus norvegicus*), considered one of the world's largest and most widely distributed rodents, are host to various zoonotic pathogens. To further understand the composition of the virus community in wild brown rats and explore new types of potentially pathogenic viruses, viral metagenomics was conducted to investigate blood, feces, and various tissues of wild brown rats captured from Zhenjiang, China. Results indicated that the composition of the virus community in different samples showed significant differences. In blood and tissue samples, members of the *Parvoviridae* and *Anelloviridae* form the main body of the virus community. *Picornaviridae*, *Picobirnaviridae,* and *Astroviridae* made up a large proportion of fecal samples. Several novel genome sequences from members of different families, including *Anelloviridae*, *Parvoviridae*, and CRESS DNA viruses, were detected in both blood and other samples, suggesting that they have the potential to spread across organs to cause viremia. These viruses included not only strains closely related to human viruses, but also a potential recombinant virus. Multiple dual-segment picornaviruses were obtained from fecal samples, as well as virus sequences from the *Astroviridae* and *Picornaviridae.* Phylogenetic analysis showed that these viruses belonged to different genera, with multiple viruses clustered with other animal viruses. Whether they have pathogenicity and the ability to spread across species needs further study.

## 1 Introduction

Infectious diseases caused by wild animal pathogens usually emerge very quickly, and some of these emerging pathogens can cause unexpected epidemics among wild animals, humans, and livestock. Wild rodents have a wide range of activities and a strong survival capacity and are common vectors of diseases affecting human life [1]. Norway rats (*Rattus norvegicus*), commonly known as brown rats, are widely found in human settlements and livestock sheds. They have intimate contact with humans and domesticated animals, posing a risk of pathogen transmission. They have the ability to spread several zoonotic pathogens, including Seoul ortho-hantavirus and cowpox virus, through direct bites, fecal and urine contamination [2,3].

Understanding the composition of wild rodent viral communities is helpful in preventing zoonotic infectious diseases. Traditional virus detection methods have more significant limitations in detecting new and unknown viruses. Viral metagenomic technologies based on second-generation sequencing technologies have been shown to have the ability to explore existing and unknown viruses in animal tissue and fecal samples [4]. Several past metagenomic studies of viruses have shown the presence of a variety of novel viruses in *Rattus norvegicus*, including potential zoonotic viruses [[5], [6], [7]]. In Norway and Hong Kong, two norovirus strains found in brown rats were similar to human norovirus [8]. Recently, members of *Hantaviridae* and *Paramyxoviridae* have been detected in wild rodents from southwest and northeast China. The results show that virus profiles of the same rodent species vary significantly in different regions [9]. Additionally, research has demonstrated that *Rattus norvegicus* feces included a variety of zoonotic enteroviruses, including astroviruses, rotaviruses, and coxsackieviruses [10]. Many zoonotic pathogens can invade blood circulation and spread to other tissues and organs to cause viremia [11,12]. An experimental team detected similar human herpes virus sequences and cross-species virulent pegivirus strains in the blood and liver of wild brown rats from southern China [13]. These results suggest that the diversity of viral communities in different tissues of wild *Rattus norvegicus* has been significantly underestimated, and viral metagenomics technology can provide strong technical support for further understanding the variety of virus communities and the discovery of new viruses.

We collected blood, brain, feces, liver, lung, oral swabs, and skin swabs from wild brown rats in Zhenjiang, eastern China, analyzed them using metagenomics and conducted phylogenetic analyses of newly identified viruses.

## 2 Materials and methods

### 2.1 Samples collection and processing

From October 2016 to April 2017, 20 adult wild brown rats were collected by trained staff of the Zhenjiang Center for Disease Control and Prevention using rat traps during pest control in Zhenjiang City, East China. The collection sites were near abandoned homes and farmland. In line with humanitarian principles, all wild brown rats were euthanized by exposure to a lethal dose of carbon dioxide. Rats over 77 mm in length were selected [14], blood was collected by cardiac puncture, and serum samples were obtained by centrifugation. After dissecting and gathering, the brain, blood, feces, liver, lung, oral swab, and skin swab samples are ground in a mortar and packed into a 1.5 ml centrifuge tube. An equal volume (500 μl) of phosphate buffered saline (PBS) was added to each centrifuge tube for resuspension, and the vortex oscillated for 5 min. After freezing and thawing three times on dry ice, supernatants were collected after centrifugation (5 min, 15,000 g) at 4 °C.

### 2.2 Viral metagenomic analysis

Twenty wild brown rats were randomly divided into four groups, each with five rats, including seven sample pools of brain, blood, feces, liver, lung, oral swabs, and skin swabs, for a total of 28 sample pools. 100 Microliters of supernatant were extracted from 5 different types of samples in each group to form a 500 μl sample pool for metagenomic analysis. Then 500 μl of supernatant was passed through a 0.45 μm filter (Millipore) to remove eukaryotic cells and bacterial-sized particles [15]. After centrifugation (13,000 g, 5 min), 166.5 μl of filtered filtrate was collected. The filtrate was treated at 37 °C with a mixture of DNases (Ambion Turbo DNase, Epicentre Baseline-ZERO), benzonase (Novagen), and RNase (Fermentas) to digest unprotected nucleic acid. Fecal samples were treated for 90 min, while other samples were treated for 1 h [16]. A QIAamp Viral RNA Mini Kit (QIAGEN) was used to extract nucleic acid according to the manufacturer's instructions, and reverse transcription was performed with six random base primers and SuperScript III reverse transcriptase (Invitrogen). The product was denatured at 95 °C for 2 min and quickly placed on ice for at least 2 min. The second strand of cDNA was then synthesized using the Klenow fragment DNA polymerase. Finally, libraries were constructed by Nextera XT DNA Sample Preparation Kit (Illumina) with dual barcoding for this pool, and the quality was evaluated using agarose gel electrophoresis. Libraries were sequenced on the MiSeq Illumina platform with 250-base paired ends using the MiSeq Reagent Kit v2 (500 cycles).

### 2.3 Bioinformatics analysis

Reads were decoded using vendor software (Illumina), and data was processed using an analytics pipeline on an analytics Linux cluster. Repeat reads and tails with low sequencing quality (Phred quality score 10 as the threshold) were deleted and clipped. The adaptors were trimmed using VecScreen's default parameters (-task blastn -reward 1 -penalty −5 -gapopen 3 -gapextend 3 -dust yes -soft_mask true - value 700 -searchsp 1,750,000,000,000), which are BLASTn parameters specifically designed for adaptor removal from the National Center for Biotechnology Information (NCBI). The cleaned reads were de novo assembled within each barcode group using the ENSEMBLE assembler, including SOAPdenovo2, ABySS, meta-Velvet, and CAP3 [17]. The assembled contigs and singlets were compared to an in-house viral proteome database using BLASTx with an E-value cutoff of < 10^−5^. Candidate viral hits are then compared to an in-house non-virus non-redundant (NVNR) protein database with an E-value cutoff of <10^−5^ to remove false-positive viral hits. The NVNR database was compiled using non-viral protein sequences extracted from the NCBI nr fasta file (based on annotation taxonomy excluding the Virus Kingdom). Contigs without significant BLASTx similarity to the viral proteome database are searched for viral protein families in the vFam database using HMMER3 [18] to detect distant viral protein similarities.

### 2.4 Viral community analysis

MEGAN software (MEtaGenome Analyzer, version 6.21.7) was used to analyze the components of these seven different virus groups [19] and to perform cluster similarity analysis and Bray-Curtis ecological distance matrix analysis with default parameters as part of the comparison option. The viral community structure and abundance results were visualized in heat maps, bar charts, a pie chart, and a bubble chart, which were generated in ChiPlot (https://www.chiplot.online/).

### 2.5 Viral sequences acquisition, phylogenetic and recombination analysis

Low sensitivity parameters were selected for de novo assembly and reference mapping of assembled and unassembled reads in the taxonomic assignment known in Geneious (version 2019.2.3) to obtain viral genomes or fragments [20]. The MUSCLE multiple sequence alignment programs in MEGA version 10.1.8 were run with default parameters to generate amino acid sequence alignments, including the sequences found in this study and closest viral relatives identified using a BLASTx search of the GenBank search and representative sequences from their respective families [21]. Phylogenetic trees were constructed using Bayesian inference (BI) in MrBayes 3.2 using mixed models and Markov chain Monte Carlo (MCMC) methods based on amino acid sequences [22]. In these analyses, the first 25% of MCMC samples were discarded as burn-in, and operations were terminated until the mean standard deviation of split frequencies was less than 0.01. Recombination analysis was performed using the Bootscan method embedded in Recombination Detection Program 5 (RDP5) [23].

### 2.6 Quality control

After the sequencing library was constructed, agarose gel electrophoresis (1% agarose) was performed to ensure consistency. All samples were processed in a biosafety cabinet and transferred using aerosol filter pipet tips to avoid cross-contamination between different sample types. All experimental materials that came into direct contact with nucleic acid samples were RNase and DNase-free, including DEPC-treated water that dissolved nucleic acids.

## 3 Results

### 3.1 Metagenomic overview of wild brown rats virome

A total of 28 libraries were constructed, and all pools were sequenced using two Illumina MiSeq channels, generating 26,939,466 raw reads with an average GC content of 48.7% (Supplementary Table S1). Of these reads, 10,329,088 (38.3%) matched viral proteins through BLASTx search based on protein sequence identity. The highest degree of matching (65.2%) was found in fecal pools, while the lowest was found in brain pools (8.7%).

After comparison, viral contigs were classified into twenty-one double-stranded DNA (dsDNA) viral families, eight single-stranded DNA (ssDNA) viral families, four double-stranded RNA (dsRNA) viral families, and sixteen single-stranded RNA (ssRNA) viral families (Fig. 1A). The majority of viral reads in blood samples belong to the Circular ssDNA family *Anelloviridae*. A majority of viral reads in brain samples were from linear ssRNA *Retroviridae* family (45.60%), along with a few from *Anelloviridae* (9.54%) and *Reoviridae* (9.69%). As fecal samples were analyzed in four libraries, viral reads related to ssDNA viruses in *Parvoviridae* (26.1%) were abundant compared to other DNA viruses such as *Papillomaviridae* and *Adenoviridae*. RNA viruses were mainly associated with the *Picobirnaviridae* and *Picornaviridae*. Bacteriophages also comprise a large part of feces, particularly *Microviridae* and *Siphoviridae*. In liver samples, eukaryotic viral reads related to *Parvoviridae* (97.3%), *Anelloviridae* (1.6%), and *Hepeviridae* (0.7%) were detected. The eukaryotic viral reads detected in lung samples included the *Anelloviridae* (3.6%), with a small number similar to the *Reoviridae*. Viruses from the *Papillomaviridae* and *Parvoviridae* acted as the most abundant part in oral and skin swabs of wild brown rats. Other families, including *Virgaviridae*, *Anelloviridae*, *Siphoviridae*, and *Microviridae,* were also present in smaller amounts in both swab samples. Using principal coordinate composition (PCoA) and correlation analysis (Fig. 1B–C) to compare viral communities of different sample types, we found that virus compositions of lung and brain were most comparable, followed by skin and oral swabs, both of which had high correlation coefficients.

**Fig. 1:**
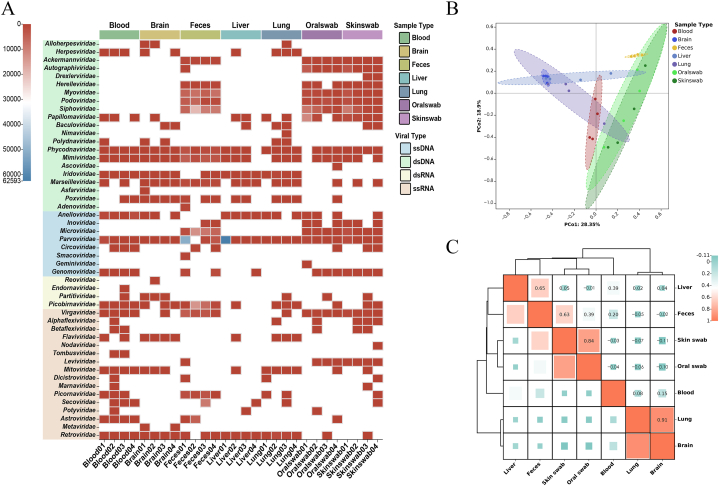
Analysis of viral taxonomy at the family level. (A) Heat map representing the read number of each viral family (unread families are represented in a blank grid). The color coding in the color legend indicates sample and viral types. (B, C) PCoA plot (B) and Correlation Clustering Heat map (C) showing similarity of viral community structures among different samples based on the Bray-Curtis ecological distance matrix. (For interpretation of the references to color in this figure legend, the reader is referred to the Web version of this article.)

A total of 1091 virus species were detected from seven different types of samples. Skin and oral swab samples had the highest number of species, 543 and 387, respectively, while only 15 were found in liver samples (Fig. 2A). Statistical results showed that 736 viral species, mostly bacteriophages, occurred only in a single sample type, mainly in skin swabs (39%) and oral swabs (25%) samples, while no endemic viruses were found in liver (Fig. 2B). Meanwhile, 33% of viral species appeared in at least two sample types, particularly *Rat minute virus* and *Rodent protoparvovirus*, distributed in all types. The bubble chart based on the five most abundant viral species in each sample type showed a high percentage of viruses in the blood, brain, feces, and lungs, ranging from 1% to 10% (Fig. 2C). It was evident from the bubble map that several skin and oral swab viruses barely appeared in other types of samples, indicating specific community characteristics of oral and skin viruses. In addition, plants and arboviruses were detected in different samples, such as *Raspberry ringspot virus*, *Chenuda virus,* and *Erythrocytic necrosis virus* [24,25].

**Fig. 2:**
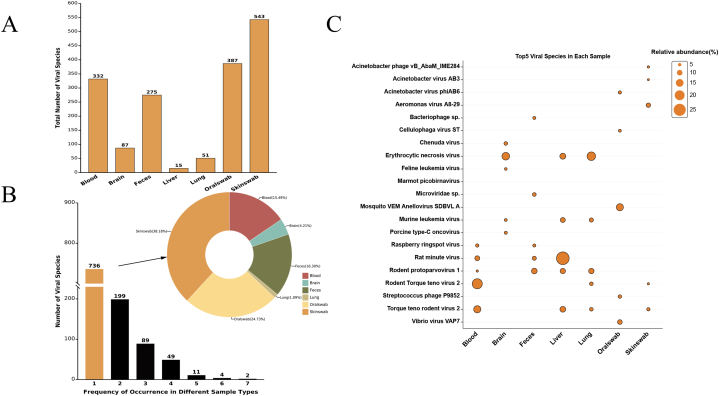
Analysis of viral taxonomy at the species level. (A) Total number of viral species in different samples. (B) The bar chart shows the number of viral species occurring at different frequencies in different samples, and the pie chart shows the distribution of endemic species in different samples. (C) The bubble plot shows the top 5 most abundant viral species of the seven sample types. The bubble size represents the relative abundance read by each species.

### 3.2 Anelloviruses

**Fig. 3:**
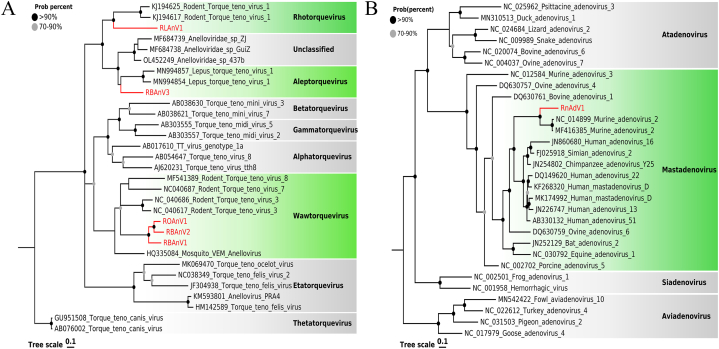
The phylogenetic analysis of *Adenoviridae* and *Anelloviridae* identified in wild *Rattus norvegicus*. (A) Phylogenetic analysis based on ORF1 amino acid sequences of anelloviruses. (B) Phylogenetic analysis based on Hexon amino acid sequences of adenoviruses. The viruses identified in this study are labeled with red branches and leaf names, and the genera assigned to them are labeled green. All other genera are marked in a grey box. Tree scales indicate amino acid substitutions per site. The size of the black dots on the nodes corresponds to bootstrap values. (For interpretation of the references to color in this figure legend, the reader is referred to the Web version of this article.)

In this study, abundant sequence reads found in different samples showed sequence similarity to the *Anelloviridae*. Five strains, including the complete open reading frame (ORF) 1 region, were identified as RBAnV1, RBAnV2, RBAnV3, RLAnV1, and ROAnV1, respectively. Except for RLAnV1, which only existed in liver samples, the other four strains were found simultaneously in multiple sample types except for fecal samples. Blast results showed that RBAnV1, RBAnV2, and ROAnV1 were closely related to rodent torque teno virus 3, showing 98.63%, 69.86%, and 97.61% amino acid identity, respectively. The length of the ORF1 region of the three is similar, 575 aa, 578 aa, and 578 aa, respectively, which is much larger than RBAnV3 (383 aa) and RLAnV1 (369 aa). Phylogenetic analysis of ORF1 amino acid sequences (Fig. 3A) showed that RBAnV1, RBAnV2, and ROAnV1 clustered in a clade belonging to *Wawtorquevirus*. RLAnV1 was inside a clade containing rodent torque teno virus 1 belonging to *Rhotorquevirus*, sharing 39.6% amino acid identity with the closest sequence (GenBank No. KJ194617). In addition, RBAnV3 was classified as *Aleptorquevirus* with 44.1% amino acid identity to torque teno virus from Lepus (GenBank No. MN994854).

### 3.3 Adenovirus

Several adenovirus sequence reads were found in fecal samples of *Rattus norvegicus*, and a complete viral hexon protein sequence of 2793 bp was obtained after assembly. Adenovirus hexon protein is a homologous trimer, which is the main capsid protein. The hexon protein-based phylogenetic tree (Fig. 3B) showed that this strain (RnAdV1) clustered with murine adenovirus 2 (GenBank No. NC014899) supported, sharing 81.4% amino acid identity.

### 3.4 CRESS DNA viruses

**Fig. 4:**
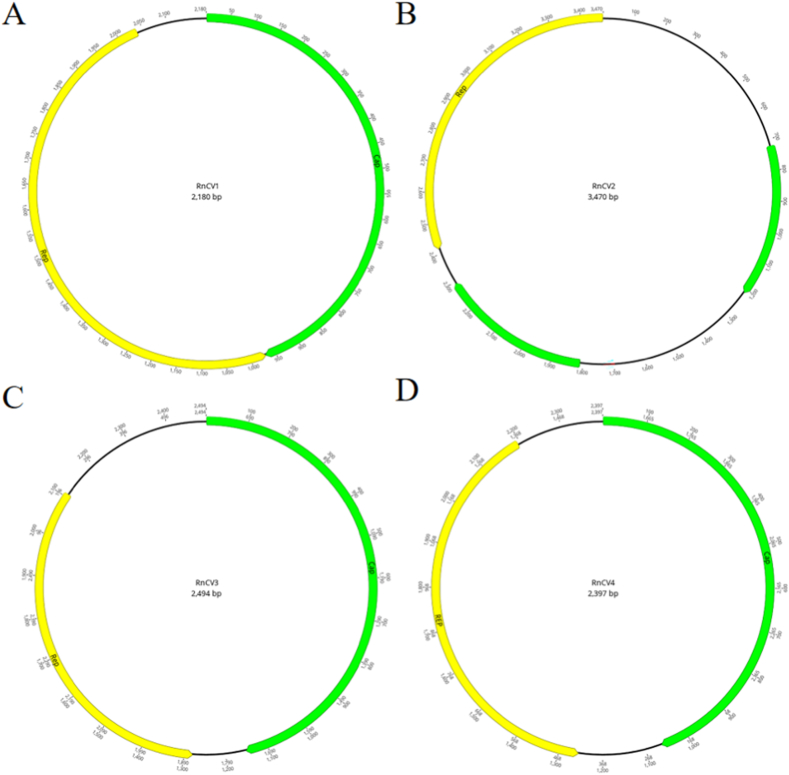
Genome structure of four CRESS DNA viruses identified in wild *Rattus norvegicus*. (A) Genome structure of RnCV1. (B) Genome structure of RnCV2. (C) Genome structure of RnCV3. (D) Genome structure of RnCV4.

Replication-associated protein (Rep)-like sequences were detected in various *Rattus norvegicus* samples, and complete genome sequences of four novel Circular replication-associated protein-encoding single-stranded (CRESS) DNA viruses were obtained. Complete circular DNA structures were identified by de novo synthesis and splicing, where the predicted Rep and other proteins were all in the opposite direction (Fig. 4). A complete genome with a length of 2180 nt (RnCV1) found in feces, skin swabs, oral swabs, and blood samples shared the highest identity with *Genomoviridae.* Blast results showed that the Rep and capsid protein (Cap) of RnCV1 were both similar to a gemykibivirus isolated from human cervicovaginal smears (GenBank No. AYN61536), showing 99.47% and 98.91% amino acid identity, respectively. Another complete genome classified as *Circoviridae,* named RnCV2, was assembled from only fecal samples. RnCV2, which was 3470 nt in length, encoded three ORFs, with one Rep and two other hypothetical proteins in opposite directions. Although the genome size and number of ORFs were different from those of traditional circoviruses, walker A motif (GxxxGK) was found in the Rep region. A smacovirus with a complete genome sequence was also found in fecal samples named RnCV3. The length of RnCV3 was 2494 nt, including 276 aa Rep and 384 aa Cap. In addition, a circovirus-like genome found in fecal samples named RnCV4 shared 81.45% Rep amino acid sequence identity with a rodent stool-associated circular virus (RodSCV) through BLASTx search. Like RodSCVs, the Rep of RnCV4 possessed conserved motifs (DRYP) but not the DDFYGW motif typical of circoviruses and contained a Rep superfamily. Interestingly, we also found a virus strain identical to a gemycircularvirus isolated from the cerebrospinal fluid of Sri Lankan patients (GenBank No. KP133076), enriching the host diversity of this virus.

**Fig. 5:**
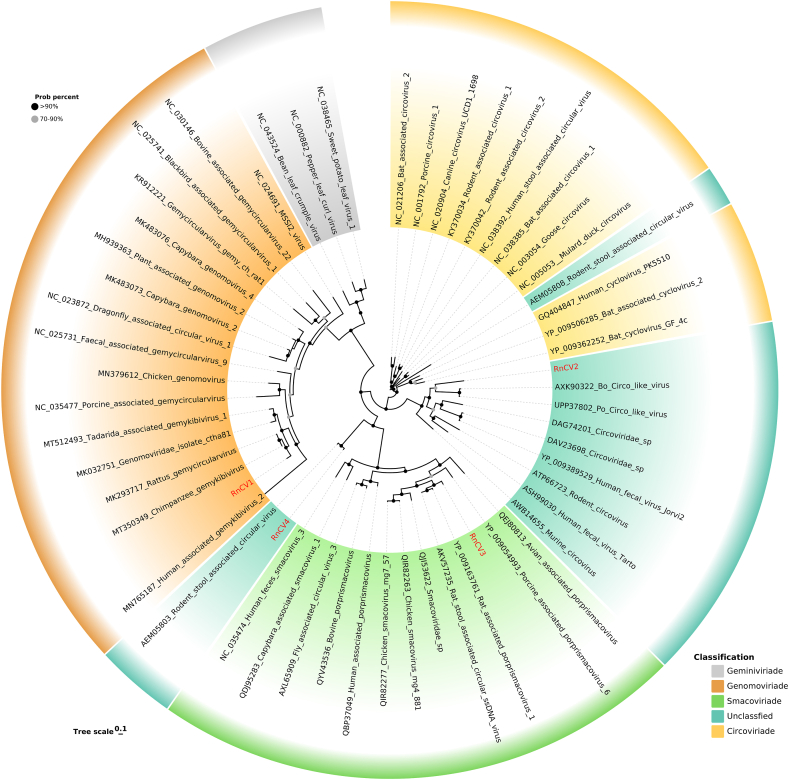
Phylogenetic analysis based on Rep protein amino acid sequences of CRESS DNA viruses. The viruses identified in this study are labeled with red branches and leave names. Tree scales indicate amino acid substitutions per site. The size of the black dots on the nodes corresponds to bootstrap values. Different taxonomic clusters were filled with different colors (see Color Legend). (For interpretation of the references to color in this figure legend, the reader is referred to the Web version of this article.)

Based on Rep proteins, phylogenetic trees (Fig. 5) were constructed and showed that RnCV1 is closely related to a chimpanzee gemykibivirus, sharing 98.9% amino acid identity. RnCV2 exhibited the closest kinship with two cicro-like viruses that originated from porcine (GenBank No. UPP37802) and bovine (GenBank No. AXK90322), shared only 34.4% and 36.1% amino acid identity, respectively. RnCV3 shared 88.2% amino acid identity with the most closely related proprismacovirus isolated from rat feces. RnCV4 clustered with a RodSCV that shared the smallest amino acid identity.

### 3.5 Parvoviruses

**Fig. 6:**
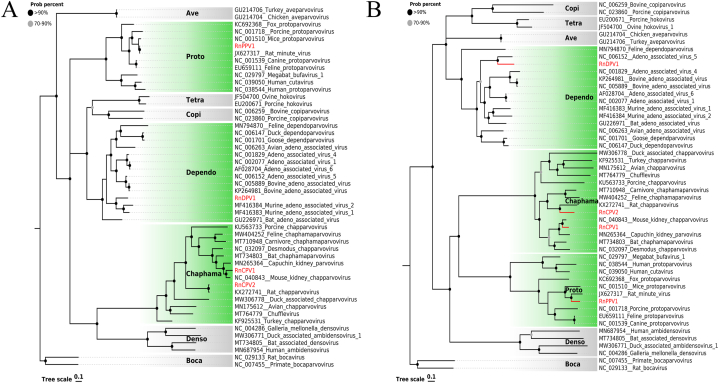
The phylogenetic analysis of NS1 and VP1 proteins for representative members of the *Parvoviridae* subfamily. (A) Phylogenetic analysis based on a single amino acid sequence of the NS1 protein of parvoviruses. (B) Phylogenetic analysis based on a single amino acid sequence of VP1 protein of parvoviruses. The viruses identified in this study are labeled with red branches and leaf names, and the genera assigned to them are labeled green. All other genera are marked in a grey box. Tree scales indicate amino acid substitutions per site. The size of the black dots on the nodes corresponds to bootstrap values. (For interpretation of the references to color in this figure legend, the reader is referred to the Web version of this article.)

Four novel parvoviruses with nearly complete genome sequences were determined from different samples (blood, feces, liver, oral swab) of *Rattus norvegicus* that included two members from *Chapparvovirus* (RnCPV1, RnCPV2), one from *Dependoparvovirus* (RnDPV1) and one from *Protoparvovirus* (RnPPV1). The length of these nearly complete genomes is 4183 nt for RnCPV1, 4178 nt for RnCPV2, 4320 nt for RnDPV1, and 4797 nt for RnPPV1. Two phylogenetic trees (Fig. 6A–B) based on protein sequences of nonstructural protein 1 (NS1) and structural protein (VP1) were constructed, respectively. Except for RnDPV1, the clustering of the other three viruses in both cases was identical. RnCPV1 and RnCPV2 clustered with two isolated rat chapparvoviruses (GenBank No. NC040843, KX272741), with which they shared 77.1% and 93.0% (NS1), 83.2% and 63.5% (VP1) amino acid identities, respectively. RnPPV1 clustered with a rat protoparvovirus, and the amino acid identity of NS1 in between was 99.9%, and VP1 was 74.7%. The results showed that RnDPV1 clustered with murine adeno-associated viruses 1 and 2 (GenBank No. MF416383, MF416384) formed a clade in the NS1 phylogenetic tree, while genotypes in the VP1 phylogenetic tree did not ultimately cluster with the same viruses but with adeno-associated virus 5 (GenBank No. NC006152). Sequence analysis using Blastx in NCBI indicated that NS1 of RnDPV1 shared the highest amino acid sequence identity (68.01%) with murine adeno-associated virus 1, which was identified in rat feces from New York. However, VP1 of RnDPV1 shared the highest amino acid identity (69.43%) with rat adeno-associated virus in *Rattus norvegicus* feces from Guangdong, China. These results indicated that RnDPV1 may be a recombinant strain.

**Fig. 7:**
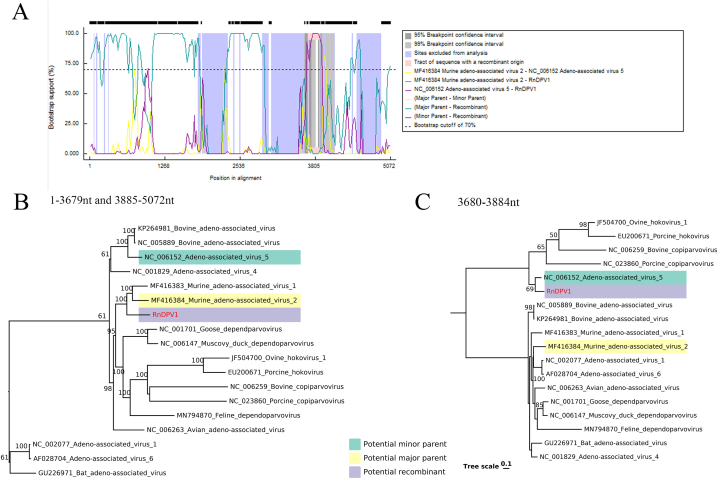
Recombinant analysis of RnDPV1. (A) BOOTSCAN proof of recombination origin based on pairwise distance, modeled with a window size 200, step size 20, and 100 Bootstrap replicates. (B, C) Phylogenetic trees were constructed based on regions 1-3679 nt, 3885-5072 nt, and exchange part 3680-3884 nt, respectively. The recombinant strain and both parental viruses were labeled with different colors (see Color Legend). (For interpretation of the references to color in this figure legend, the reader is referred to the Web version of this article.)

Potential parent sequences and potential recombination sites were identified using RDP 5.0, showing two recombination events (Fig. 7A). The murine adeno-associated virus 2 (GenBank No. MF416384) was the major parent, and the minor parent was adeno-associated virus 5 (GenBank No. NC006152), both found in American rats. The confirmation table showed that RnDP1, with a recombinant score of 0.614, was almost certainly a recombinant strain. Phylogenetic tree based on the region of 1–3679 nt, 3885–5072 nt, and exchange part 3680–3884 nt (Fig. 7B–C) showed that RnDP1 clustered with two parents in different trees, which further confirmed recombination events.

### 3.6 Astroviruses

**Fig. 8:**
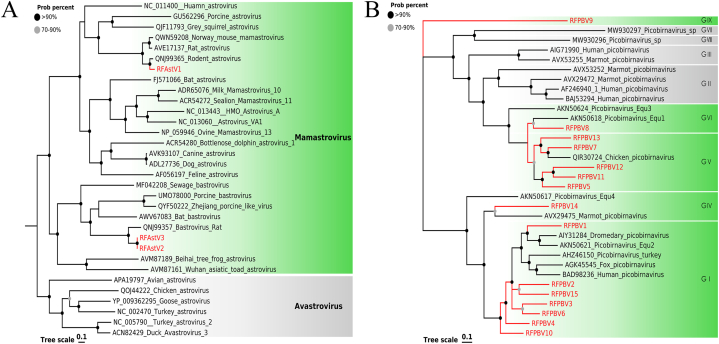
The phylogenetic analysis of *Astroviridae* and *Picobirnaviridae* identified in wild *Rattus norvegicus*. (A) Phylogenetic analysis based on the amino acid sequences of RdRp protein of astroviruses. (B) Phylogenetic analysis based on the amino acid sequences of RdRp of picobirnaviruses. The viruses identified in this study are labeled with red branches and leaf names, and the genera assigned to them are labeled green. All other genera are marked in a grey box. Tree scales indicate amino acid substitutions per site. The size of the black dots on the nodes corresponds to bootstrap values. (For interpretation of the references to color in this figure legend, the reader is referred to the Web version of this article.)

In fecal samples, three nearly complete astrovirus genome sequences were detected in this study, named RFAstV1, RFAstV2, and RFAstV3. The genome of RFAstV1 was 6085 bp in length and contained three complete ORFs, ORF1a (744 aa), ORF1b (473 aa), and ORF2 (745 aa). The nearly complete genomes of RFAstV2 and RFAstV3 were 5853 bp and 5736 bp in length, both consisting of two ORFs that encoded a nonstructural protein, including RNA-dependent RNA polymerase (RdRp) and a structural protein. The length of nonstructural proteins was 1246 aa, 1212 aa, and the length of structural proteins was 679 aa. Conserved domains analysis indicated that RFAstV1 ORF1a has a trypsin-like serine protease domain at 401–521 aa, while the RNA methyltransferase domain was found in both RFAstV2 and RFAstV3. Phylogenetic analysis based on amino acid sequences of the RdRp protein (Fig. 8A) showed that RFAstV1 was associated with a rodent astrovirus (Genbank No. QNJ99365), shared 84.62% amino acid sequence identity of the RdRp. RFAstV2 and RFAstV3 clustered with rat bastrovirus (GenBank No. QNJ99357), and the amino acid sequence identities of the RdRp were 81.82% and 81.73%, respectively.

### 3.7 Picobirnaviruses

This study identified a total of 15 strains containing the RdRp domain and 13 strains containing capsid sequences in *Rattus norvegicus* feces. These RdRp shared 53.50%–81.69% amino acid identities with those of other known picobirnavirus (PBV), most of which showed amino acid identities of less than 80%. The amino acid identities of capsid proteins were less than 45%, and the lowest was only 27.58%. Phylogenetic trees were constructed based on RdRp proteins and known genotypes (GI-GVIII) from different hosts (Fig. 8Β). The tree showed that a strain that shared the lowest identity with other PBVs formed a separate branch. This result suggested that this strain represented a new genotype classified as GIX. Most of the remaining strains were classified as GI and GV genotypes, and a few strains were scattered among other genotypes. These results fully demonstrated the high diversity of PBVs.

### 3.8 Picornaviruses

**Fig. 9:**
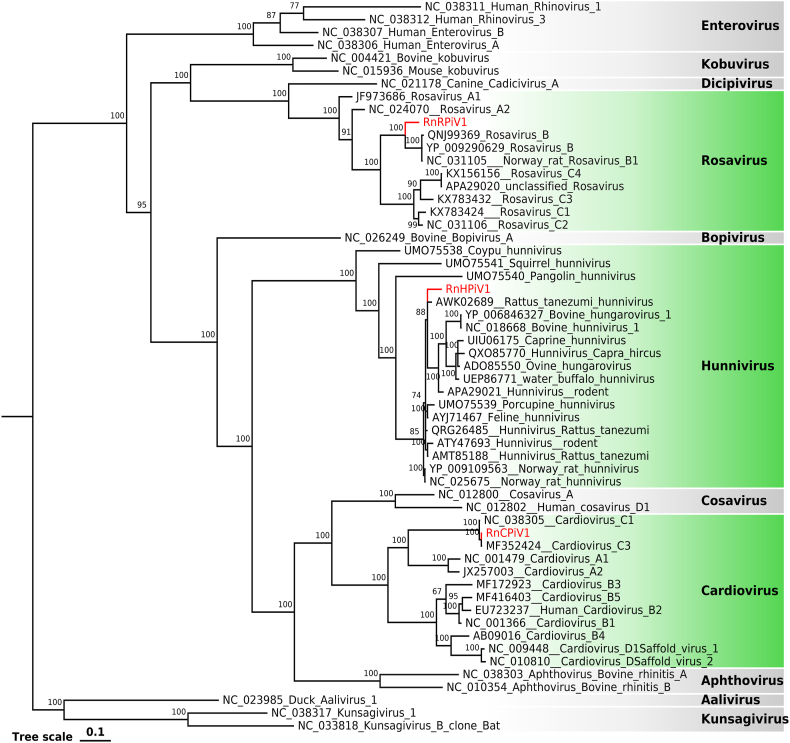
The phylogenetic analysis of *Picornaviridae* identified in wild *Rattus norvegicus*. Phylogenetic analysis based on amino acid sequences of 3D polymerase protein of picornaviruses. The viruses identified in this study are labeled with red branches and leaf names, and the genera assigned to them are labeled green. All other genera are marked in a grey box. Tree scales indicate amino acid substitutions per site. The size of the black dots on the nodes corresponds to bootstrap values. (For interpretation of the references to color in this figure legend, the reader is referred to the Web version of this article.)

One cardiovirus (RnCPiV1) was identified in fecal samples of wild brown rats, which showed 93.88% amino acid identity across the polyprotein to cardiovirus C. RnCPiV1 had a genome size of 8772 bp, containing a single ORF encoding a 2322 amino acid polyprotein consisting of a leader peptide (L), four structural proteins (VP1, VP2, VP3, and VP4), and seven nonstructural proteins (2A, 2B, 2C, 3A, 3B, 3C, 3D). Phylogenetic analysis based on 3D polymerase proteins (Fig. 9) showed that RnCPiV1 clustered with cardiovirus C3, which shared 86.3 and 99.0% amino acid identities in their P1 and P2 + P3 regions, respectively. A new species of cardiovirus should have a P1 amino acid identity of < 60% when compared to other known cardiovirus species, according to the International Committee on Taxonomy of Viruses (ICTV). However, RnCPiV1 failed to meet the criteria but may belong to a novel type within the cardiovirus C species.

Rodent stool-associated picornavirus (Rosavirus) is a newly discovered novel virus that exists only in mice and currently includes three types (A, B, C) [26]. In this study, we found a novel rosavirus in fecal samples named RnRPiV1. The complete genome of RnRPiV1 consisted of 9177 bp with a GC content of 51.0%. The genome has a similar structural composition to other rosaviruses, with a large reading frame that encodes a polyprotein divided into P1, P2, and P3 regions. Meanwhile, the 3D polymerase protein also contained KDE [LI]R, GG [LMN]PSG, YGDD, and FLKR motifs, in which the second Gly was replaced as other rosavirus B by Ala in GG [LMN]PSG. The phylogenetic placement (Fig. 9) showed that RnRPiV1 is a member of rosavirus B species, which shared 70.6%–71.2%, 76.8%–77.1% amino acid identities in the P1, P2 + P3 region with other rosavirus B viruses.

Another nearly full-length hunnivirus genome sequence, belonging to a genus within the *Picornaviridae* family, was recently established in 2013 [27]. The sequence in this study (RnHPiV1) was 7044 bp and included an incomplete ORF of 6504 nt encoding polyprotein. Comparison results using BLASTx showed that RnHPiV1 shared 63.35%–98.03%, 78.20%–97.07%, and 82.03%–97.92% amino acid identities with rat-derived hunniviruses in the P1, P2, and P3 regions, respectively. To further analyze the genetic relationship of RnHPiV1 with other hunniviruses, phylogenetic analysis (Fig. 9) showed that RnHPiV1 formed a separate branch.

## 4 Discussion

Expansion of human settlements and exposure to wild hosts may increase the number of rat-borne diseases and the risk of infection [28,29]. The recent outbreak of the zoonotic monkeypox virus, whose main host is wild rodents, can be transmitted to humans through close contact, and there has been significant human-to-human transmission [30]. Wild brown rats that host a variety of viruses, live in areas close to residential areas and farmland, posing challenges to the prevention and control of infectious diseases [31].

This study aimed to analyze and compare the viral community characteristics of different wild *Rattus norvegicus* samples using viral metagenomics technology to further enrich virus diversity. Viral metagenomics can map host viral diversity and help identify zoonotic and emerging viruses. Sequences closely and remotely related to known viral sequences and many sequences of unknown taxonomic origin were identified. We detected more than 50 virus sequences from 49 families in 28 libraries, including some viruses that can cause severe infection in animals and humans. Another metagenomic analysis of rodent viruses from two border provinces in northeast and southwest China showed that 10 virus families were detected in *Rattus norvegicus* [9]. The virus families labeled in this study had higher diversity and overlayed with the community composition of *Rattus norvegicus* from other provinces in China (7/49). The virus composition of *Rattus norvegicus* in the Zhenjiang area was dominated by *Parvoviridae* and *Picornaviridae*, but no *Hantaviridae* was detected, which was similar to the enterovirus community composition of *Rattus norvegicus* in Berlin [32]. The composition of viruses of *Rattus norvegicus* in different regions of China is quite different, which may be influenced by human activities and the living environment. In addition, we also detected a small number of plant viruses and insect viruses, which may be from diet or environmental pollution. The composition of viruses in different samples showed variability, and most of the virus species showed organ specificity, but some viruses existed in multiple samples, with a wide range of organ distribution abilities. Interestingly, the liver had the fewest virus species, which like the blood samples consisted mainly of *Parvoviridae* and *Anelloviridae*. As in another metagenomic-based study of urban rat liver viruses, we detected a high abundance of torque teno virus (TTV), which is thought to be associated with liver disease, but we did not detect other sequences associated with the underlying etiology of hepatitis [13]. The unusually high number of reads in a single pool may be because sample sizes for different libraries are determined according to the relative luminance of the electrophoretic bands. In addition, the number of wild *Rattus norvegicus* collected by us is limited, and the area is relatively concentrated, so the results may be biased, which may be improved by expanding the sampling area and quantity in the future.

Various mammals and humans can be infected with anelloviruses, which are highly genetically diverse [33,34]. Five strains of anelloviruses belonging to three different genera were detected in blood, tissue, and organ samples but not fecal samples. Although previous experimental results have supported the possibility of fecal-oral transmission of anelloviruses [35], the dietary source cannot be completely ruled out. The three strains RBAnV1, RBAnV2, and ROAnV1, belonging to the genus *Wawtorquevirus,* showed apparent homology with known rodent viruses at amino acid level, and the ORF1 region length was similar to that of human anelloviruses. We detected RBAnV1 and RBAnV2 in blood, liver, lung, and skin swabs of the same wild *Rattus norvegicus*, indicating that multiple viral genotypes can be seen from the same wild *Rattus norvegicus* in a specific region or time and can be found in the blood and different tissues in its body, which may have the ability to cause viremia. These characteristics are similar to those of anellovirus infection in wild rodents in the UK, and all have common features with anellovirus infection in humans [36]. All the anelloviruses found in this study cluster with rodent TTV, and their relative abundance in the liver is high, indicating that they may be hepatophilic. There have been studies showing that TTV replicates in the liver and exists in cross-species transmission [13]. Confirmation of pathogenicity and cross-species transmission of the five anelloviruses reported in this experiment is still required.

It has been shown that adenoviruses are capable of causing cancer in rodents or of transforming rodent cells in vitro [37]. Adenoviruses are widely distributed and may be responsible for respiratory and gastrointestinal diseases in humans as well [38]. The adenovirus fragment obtained in this study was too short to be further analyzed and could only be roughly classified as murine adenovirus type 2.

The genome length of the five strains of CRESS DNA viruses we obtained ranged from 2.1 kb to 3.4 kb, and they belonged to four different families, showing high genetic diversity. In 2015, a new strain of gemycircularvirus was identified by deep sequencing the cerebrospinal fluid of patients with encephalitis of unknown cause in Sri Lanka, while related genomes were present in feces from diarrhea cases of unknown origin in Nicaragua and Brazil, and raw sewage from Nepal [39]. The same virus sequence was found in the feces of a wild *Rattus norvegicus*. Considering that the Zhenjiang sampling site is located in the Yangtze River basin, and other places where the virus was found are also located in coastal areas, it is highly likely that the virus was transmitted through water contamination. In addition, we found an entirely new strain of gemykibivirus (RnCV1) in the blood of wild brown rats with similarities to viruses found in bats, pigs, chickens, dragonflies, and even humans that have been isolated from many types of human tissue [40]. Although identifying RnCV1 in blood samples supports the possibility of replication in rodent hosts, experiments to confirm this idea still need to be completed. Phylogenetic analysis showed that RnCV2 was closely related to both circovirus strains in domestic animals in China. Given that CRESS DNA viruses have a wide range of hosts and are associated with various infections, particularly gastrointestinal diseases in pigs [41], RnCV2 may potentially spread across species. Further studies are needed to determine the cellular host and transmissibility of these viruses.

Parvoviruses were detected in all sample pools and consisted of many viral reads, especially in the liver, which showed high abundance. In the human liver, parvovirus infection is often associated with acute liver injury [42]. A dependoparvovirus (RnDPV1) with low similarity to other murine parvoviruses formed different branches on the two phylogenetic trees, suggesting potential recombination. Further analysis confirmed recombination events, and both parental viruses were found in rodent feces in the United States, implying that rodent migration may promote recombination. Two of RnDPV1's parent viruses, adeno-associated viruses (AAV) type 2 and type 5, have been shown to be able to be isolated from humans. In liver studies, various stereotypes of AAV are hepatotropic after intravenous administration in mice [43]. Parvoviruses can usually interact with multiple viruses [44,45], so the effect of recombination should be fully considered when considering the diversity of parvoviruses.

Astroviruses have a high genetic diversity, and different genotypes can be found in the same animal [46,47]. According to a study investigating the variety of rodent astroviruses in other regions of southern China, 11.9% of rodents carried astroviruses [48]. We obtained the complete genome sequences of three astroviruses from the feces of wild *Rattus norvegicus*, which clustered in two distinct clusters and were distantly related.

PBVs accounts for a large proportion of wild brown rats enterovirus. A total of 28 new PBV strains were obtained from fecal samples of wild *Rattus norvegicus*, indicating that *Rattus norvegicus* is one of the critical wildlife reservoirs for PBV. Two studies on the diversity of RNA viruses in wild animals in the plateau region of western China showed that a large number of segmented PBVs and a small number of unsegmented PBVs were found in the feces of Tibetan rats and Himalayan zoko rats, which were generally consistent with PBVs in woodchuck [49,50]. Most PBVs in this experiment were grouped with known genotypes but clustered with other animals. In addition, a single PBV was clustered, forming a deep clade that may represent a new PBV gene group. This situation indicates that environmental factors have an important influence on PBV evolution. We did not find unsegmented PBV, which prevented us from further investigating the possibility of PBV recombination. Although PBV has been detected in human and various mammalian feces, its role in disease remains unclear [51]. However, we believe PBV should be widespread in wild rodents in China.

Picornaviruses have a strong ability to mutate, and with the development of research, more and more new picornaviruses have been discovered in various previously unknown hosts [[52], [53], [54]]. Wild rodents have been shown to host a variety of picornaviruses, similar to a report on *Rattus norvegicus* in New York City, the *Picornaviridae* in this study showed high abundance and diversity in fecal samples [55]. Cardiovirus is a genus of the *Picornaviridae*, mainly divided into three species: A, B, and C. Except for type B, which has been shown to cause human disease, the other two types have only been found in rodents. Phylogenetic analysis showed that RnCPiV1 belonged to type C, and its closest relative was detected in northern China. In addition, a strain of rosavirus was detected in feces, classified as rosavirus B. Previous experimental results showed that cardiovirus C and rosavirus B existed only in *Rattus norvegicus*, and both new strains in this study also did not establish close relationships with other species. Another hunnivirus (RnHPiV1) was also detected in feces, and phylogenetic analysis showed that it was the closest to a virus detected in *Rattus tanezumi* but far from *Rattus norvegicus*. This result was different from a previous study on rodent hunnivirus diversity in southern China [56], RnHPiV1 may be a potentially novel strain.

*Rattus norvegicus* is widely distributed in rural and urban areas of developing countries and may be an important source of many zoonotic pathogens [57,58]. With the expansion of human habitation and the development of animal husbandry, opportunities for contact between wild *Rattus norvegicus* and humans and domestic animals are also increasing, providing opportunities for the spread of new and recurrent diseases. In wild adult *Rattus norvegicus*, we discovered many unknown viruses from different families. These included viruses closely related to humans and viruses with recombinant and cross-species transmission potential, showing high genetic diversity in a limited surveillance area and a single rodent species. However, we cannot rule out the possibility that these viruses originate from foodborne contamination, and further research is needed to determine whether they are contagious or pathogenic.

## Data availability

The datasets presented in this study can be found in online repositories. The names of the repository/repositories and accession number(s) can be found in the article/Supplementary material.

## Ethics statement

All animal experiments were conducted in accordance with the Guidelines for Experimental Animals of the Ministry of Science and Technology (Beijing, China) and an ethical approval given by Ethics Committee of Jiangsu University (approval No. UJS2020088).

## Authors contributions

**Zi Zhuang**: performed the experiments, analyzed and interpreted the data, wrote the paper. **Lingling Qian**: analyzed and interpreted the data. **Juan Lu**: performed the experiments. **Xiaodan Zhang**: contributed reagents, materials, analysis tools or data. **Asif Mahmood**: analyzed and interpreted the data. **Lei Cui**: contributed reagents, materials, analysis tools or data. **Huiying Wang**: contributed reagents, materials, analysis tools or data. **Xiaochun Wang**: contributed reagents, materials, analysis tools or data. **Shixing Yang**: contributed reagents, materials, analysis tools or data. **Likai Ji**: analyzed and interpreted the data. **Tongling Shan**: conceived and designed the experiments. **Quan Shen**: conceived and designed the experiments. **Wen Zhang**: conceived and designed the experiments.

## Declaration of competing interest

The authors declare that they have no known competing financial interests or personal relationships that could have appeared to influence the work reported in this paper.

## Figures and Tables

**figs1:**
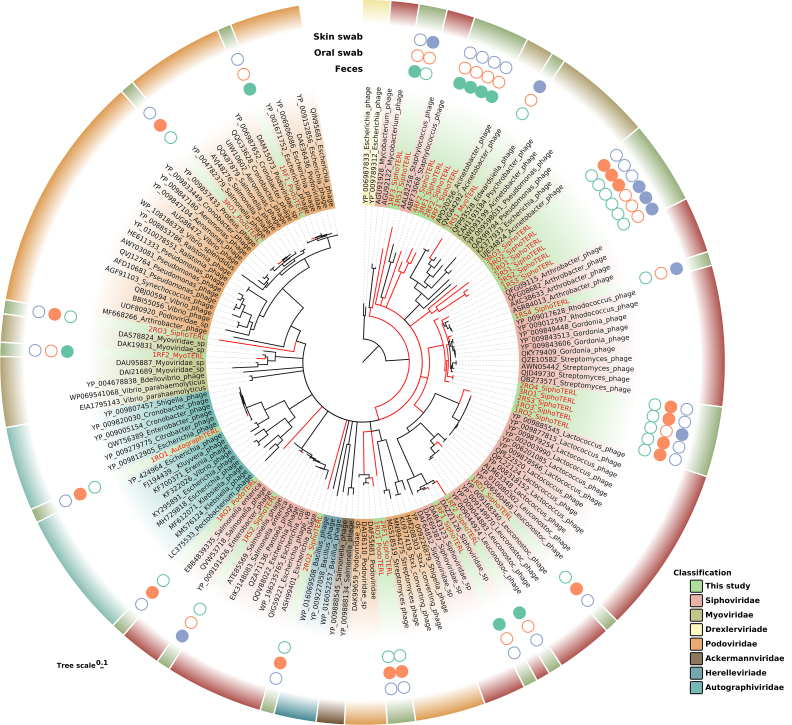
Phylogenetic analysis based on TerL amino acid sequences of *Caudovirales*. The viruses identified in this study are labeled with red branches and leave names. Tree scales indicate amino acid substitutions per site. The size of the black dots on the nodes corresponds to bootstrap values. Different taxonomic clusters were filled with different colors (see Color Legend). Peripheral binaries show colored circles in which strain samples were identified.

**figs2:**
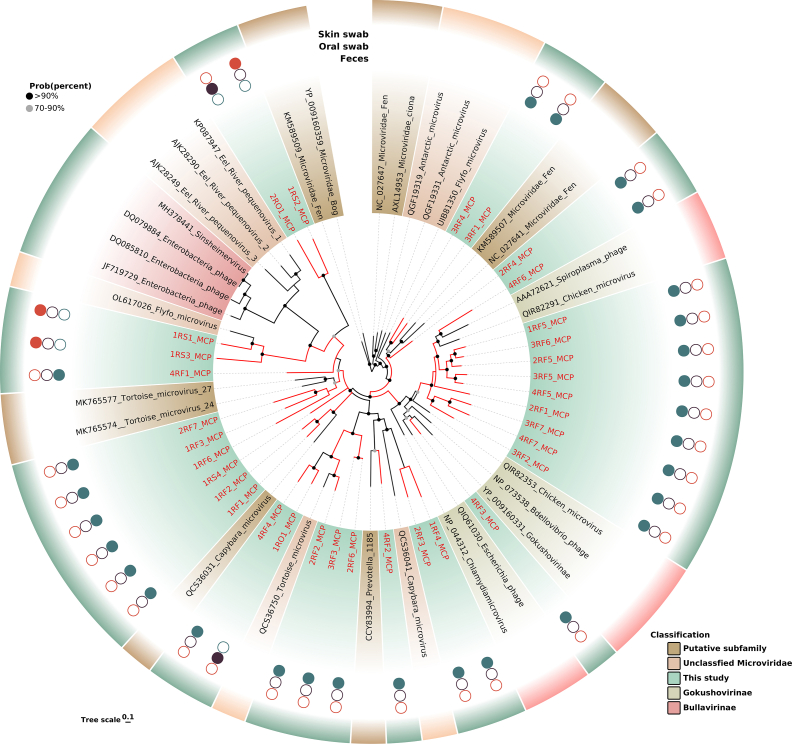
Phylogenetic analysis based on MCP amino acid sequences of microviruses. The viruses identified in this study are labeled with red branches and leave names. Tree scales indicate amino acid substitutions per site. The size of the black dots on the nodes corresponds to bootstrap values. Different taxonomic clusters were filled with different colors (see Color Legend). Peripheral binaries show colored circles in which strain samples were identified.
