# Supplementary material for: Comparison of viral communities in the blood, feces and various tissues of wild brown rats (Rattus norvegicus)
Source: Heliyon. 2023 Jun 13;9(6):e17222. doi: 10.1016/j.heliyon.2023.e17222 (PMC10300334; doi:10.1016/j.heliyon.2023.e17222)
Supplement: Multimedia component 1 [file mmc1.docx]

**The most abundant phages in** **feces, oral swabs, and skin swabs of *Rattus norvegicus***

Numerous sequences showing significant similarity to phages were detected in feces, oral swabs, and skin swabs of *Rattus norvegicus*, mainly belonging to the *Caudovirales* order and the *Microviridae* family. Fifty-five phage-related viral sequences were obtained, consisting of members of the families *Siphoviridae* (n=27), *Podoviridae* (n=4), *Myoviridae* (n=2), *Autographviridae* (n=1), and *Microviridae* (n=33). These sequences all contained virus hallmark genes (conserved domains) of each virus group, including genes encoding phage terminate large subunits (TerL) for *Caudovirales* and major capsid protein (MCP) for *Microviridae*. The microviruses identified in this study were the most abundant phages in fecal samples. Most phages classified as *Caudovirales* came from oral and skin swab samples, particularly the *Siphoviridae* family. In addition, 14 complete genome sequences of microviruses were obtained, ranging in size from 4254 to 6367 nt. BLASTx results showed that these sequences shared sequence identities with their best matches ranging from 32.72% to 100%.

Due to most sequences belonging to the *Caudovirales* order and the *Microviridae* family, highly conserved evolutionary TerL and MCP-based phylogenetic trees were constructed, respectively. The first tree (Fig. S1) showed that the vast majority of TerL sequences in this study were clustered in their respective known families, and no branches drifted away from the virus group. The *Microviridae* family included two approved subfamilies, *Gokushovirinae* and *Bullavirinae*, found in various animal gut and fecal samples. The second MCP-based phylogenetic tree (Fig. S2) showed eleven sequences identified in this study belonging to the *Gokushovirinae* subfamily. The rest of the sequences are scattered, occurring in multiple putative subfamilies and branches of unclassified *Microviridae*. The results of the analysis showed that the overwhelming majority of phages from oral and skin swabs clustered together. There was a high amino acid similarity between the bacteriophages found in this experiment and the bacteriophages identified in the feces of chronically ill human patients, as with human intestinal phages, sequences in this study from the diversity of the *Caudovirales* order and the *Microviridae* family form somewhat stable aggregates. Further research will focus on analyzing phage characteristic gene functions and drug resistance.
