# Supplementary material for: Comparison of viral communities in the blood, feces and various tissues of wild brown rats (Rattus norvegicus)
Source: Heliyon. 2023 Jun 13;9(6):e17222. doi: 10.1016/j.heliyon.2023.e17222 (PMC10300334; doi:10.1016/j.heliyon.2023.e17222)
Supplement: Multimedia component 2 [file mmc2.docx]

Supplementary Table S1 The summary of library information of wild brown rats

| Library ID | Sample type | Sample source | Sample site | Collecting Date | # of Sample | # of Unique reads | NCBI SRA accession no. |
| --- | --- | --- | --- | --- | --- | --- | --- |
| Blood01 | Blood | Wild brown rat(Rattus norvegicus) | Zhenjiang | 2016-2017 | 5 | 133434 | SRR19628311 |
| Blood02 | Blood | Wild brown rat(Rattus norvegicus) | Zhenjiang | 2016-2017 | 5 | 87107 | SRR19628967 |
| Blood03 | Blood | Wild brown rat(Rattus norvegicus) | Zhenjiang | 2016-2017 | 5 | 164889 | SRR19629385 |
| Blood04 | Blood | Wild brown rat(Rattus norvegicus) | Zhenjiang | 2016-2017 | 5 | 112995 | SRR19629444 |
| Brain01 | Brain | Wild brown rat(Rattus norvegicus) | Zhenjiang | 2016-2017 | 5 | 171630 | SRR19629467 |
| Brain02 | Brain | Wild brown rat(Rattus norvegicus) | Zhenjiang | 2016-2017 | 5 | 120734 | SRR19629667 |
| Brain03 | Brain | Wild brown rat(Rattus norvegicus) | Zhenjiang | 2016-2017 | 5 | 90498 | SRR19629510 |
| Brain04 | Brain | Wild brown rat(Rattus norvegicus) | Zhenjiang | 2016-2017 | 5 | 139640 | SRR19629666 |
| Feces01 | Feces | Wild brown rat(Rattus norvegicus) | Zhenjiang | 2016-2017 | 5 | 1835145 | SRR7957435 |
| Feces02 | Feces | Wild brown rat(Rattus norvegicus) | Zhenjiang | 2016-2017 | 5 | 1019788 | SRR7957436 |
| Feces03 | Feces | Wild brown rat(Rattus norvegicus) | Zhenjiang | 2016-2017 | 5 | 1823190 | SRR7957437 |
| Feces04 | Feces | Wild brown rat(Rattus norvegicus) | Zhenjiang | 2016-2017 | 5 | 1333336 | SRR7957438 |
| Liver01 | Liver | Wild brown rat(Rattus norvegicus) | Zhenjiang | 2016-2017 | 5 | 230745 | SRR19630045 |
| Liver02 | Liver | Wild brown rat(Rattus norvegicus) | Zhenjiang | 2016-2017 | 5 | 205118 | SRR19630085 |
| Liver03 | Liver | Wild brown rat(Rattus norvegicus) | Zhenjiang | 2016-2017 | 5 | 143082 | SRR19630008 |
| Liver04 | Liver | Wild brown rat(Rattus norvegicus) | Zhenjiang | 2016-2017 | 5 | 18256 | SRR19630086 |
| Lung01 | Lung | Wild brown rat(Rattus norvegicus) | Zhenjiang | 2016-2017 | 5 | 28092 | SRR19630087 |
| Lung02 | Lung | Wild brown rat(Rattus norvegicus) | Zhenjiang | 2016-2017 | 5 | 1101783 | SRR19630088 |
| Lung03 | Lung | Wild brown rat(Rattus norvegicus) | Zhenjiang | 2016-2017 | 5 | 683960 | SRR19631887 |
| Lung04 | Lung | Wild brown rat(Rattus norvegicus) | Zhenjiang | 2016-2017 | 5 | 116790 | SRR19631888 |
| Oralswab01 | Oralswab | Wild brown rat(Rattus norvegicus) | Zhenjiang | 2016-2017 | 5 | 136518 | SRR7957439 |
| Oralswab02 | Oralswab | Wild brown rat(Rattus norvegicus) | Zhenjiang | 2016-2017 | 5 | 151882 | SRR7957476 |
| Oralswab03 | Oralswab | Wild brown rat(Rattus norvegicus) | Zhenjiang | 2016-2017 | 5 | 72794 | SRR7957477 |
| Oralswab04 | Oralswab | Wild brown rat(Rattus norvegicus) | Zhenjiang | 2016-2017 | 5 | 23234 | SRR7957493 |
| Skinswab01 | Skinswab | Wild brown rat(Rattus norvegicus) | Zhenjiang | 2016-2017 | 5 | 228666 | SRR7958012 |
| Skinswab02 | Skinswab | Wild brown rat(Rattus norvegicus) | Zhenjiang | 2016-2017 | 5 | 67072 | SRR7958143 |
| Skinswab03 | Skinswab | Wild brown rat(Rattus norvegicus) | Zhenjiang | 2016-2017 | 5 | 49428 | SRR7958198 |
| Skinswab04 | Skinswab | Wild brown rat(Rattus norvegicus) | Zhenjiang | 2016-2017 | 5 | 35329 | SRR7958200 |
