# Supplementary material for: Comparison of viral communities in the blood, feces and various tissues of wild brown rats (Rattus norvegicus)
Source: Heliyon. 2023 Jun 13;9(6):e17222. doi: 10.1016/j.heliyon.2023.e17222 (PMC10300334; doi:10.1016/j.heliyon.2023.e17222)
Supplement: Multimedia component 3 [file mmc3.docx]

| Virus strain ID | GenBank accession no.  Supplementary Table S2 The sequence numbers of viruses in different libraries of this study. | Isolation source | ID of affiliated Library | Genome/Contig size (nt) | Accession no.of best matched virus | Organism | Coverage(%) | Identity(%) |
| --- | --- | --- | --- | --- | --- | --- | --- | --- |
| RnAdV1 | ON907850 | feces | Feces01 | 3203 | AWB14652 | Murine adenovirus 2 | 86 | 81.31 |
| RBAnV1 | ON907851 | blood | Blood01 | 2546 | AVE14390 | Rodent Torque teno virus 3 | 60 | 98.63 |
| RBAnV2 | ON907852 | blood | Blood04 | 2233 | QWK51327 | Rodent Torque teno virus 3 | 83 | 69.86 |
| RBAnV3 | ON907853 | blood | Blood03 | 2037 | UJQ88104 | Anelloviriade sp. | 46 | 61.81 |
| RLAnV1 | ON907854 | liver | Liver01 | 1658 | QYD02305 | Torque teno felis virus | 55 | 44.13 |
| ROAnV1 | ON907855 | oralswab | Oralswab01 | 2254 | QZE11939 | Rodent Torque teno virus | 62 | 99.35 |
| RFAstV1 | ON907856 | feces | Feces01 | 6085 | QNJ99365 | Rodent astrovirus | 99 | 88.51 |
| RFAstV2 | ON907857 | feces | Feces02 | 5853 | QNJ99356 | Bastrovirus Rat | 99 | 90.85 |
| RFAstV3 | ON907858 | feces | Feces02 | 5736 | QNJ99356 | Bastrovirus Rat | 99 | 91.21 |
| RnCV1 | ON907859 | blood | Blood04 | 2180 | AYN63516 | Human associated gemykibivirus 2 | 89 | 99.47 |
| RnCV2 | ON907860 | feces | Feces02 | 3470 | QJQ33756 | CRESS virus sp. | 80 | 40.78 |
| RnCV3 | ON907861 | feces | Feces01 | 2494 | QTZ20023 | Rat stool-associated circular ssDNA virus | 99 | 89.13 |
| RnCV4 | ON907862 | skinswab | Skinswab01 | 2397 | AEM05803 | Rodent stool-associated circular genome virus | 95 | 81.45 |
| RnCPV1 | ON907863 | skinswab | Skinswab01 | 4183 | AWB14583 | Murine chapparvovirus | 47 | 77.69 |
| RnCPV2 | ON907864 | feces | Feces03 | 4178 | YP_010086594 | Rat parvovirus 2 | 46 | 92.97 |
| RnDPV1 | ON907865 | blood | Blood01 | 4320 | YP_010086819 | Murine adeno-associated virus 1 | 99 | 68.01 |
| RnPPV1 | ON907866 | liver | Liver01 | 4797 | AAM93274 | Kilham rat virus | 99 | 96.97 |
| RnCPiV1 | ON907867 | feces | Feces01 | 8772 | AWK02683 | cardiovirus C3 | 80 | 93.88 |
| RnRPiV1 | ON907868 | feces | Feces03 | 9177 | AOQ26201 | Rosavirus B | 99 | 73.42 |
| RnHPiV1 | ON907869 | feces | Feces03 | 7044 | AWK02689 | Rattus tanezumi hunnivirus | 99 | 97.63 |
| RFPBV1 | ON907870 | feces | Feces02 | 1620 | UAW00832 | Porcine picobirnavirus | 99 | 64.01 |
| RFPBV2 | ON907871 | feces | Feces02 | 1641 | QXV86678 | Picobirnavirus sp. | 99 | 66.18 |
| RFPBV3 | ON907872 | feces | Feces02 | 1602 | UBJ26204 | Rodent associated picobirnavirus | 99 | 73.31 |
| RFPBV4 | ON907873 | feces | Feces03 | 1305 | UAW00634 | Porcine picobirnavirus | 98 | 77.75 |
| RFPBV5 | ON907874 | feces | Feces03 | 1584 | UAW00341 | Porcine picobirnavirus | 98 | 65.39 |
| RFPBV6 | ON907875 | feces | Feces03 | 1602 | UBJ26204 | Rodent associated picobirnavirus | 99 | 77.44 |
| RFPBV7 | ON907876 | feces | Feces03 | 1500 | QXV86690 | Picobirnavirus sp. | 97 | 81.69 |
| RFPBV8 | ON907877 | feces | Feces04 | 1599 | AVX53294 | Marmot picobirnavirus | 99 | 80.34 |
| RFPBV9 | ON907878 | feces | Feces01 | 1890 | UDL14560 | Sichuan picobirnavirus B3 | 99 | 64.39 |
| RFPBV10 | ON907879 | feces | Feces03 | 1470 | UAW00565 | Porcine picobirnavirus | 99 | 80.51 |
| RFPBV11 | ON907880 | feces | Feces02 | 1290 | QXV86700 | Picobirnavirus sp. | 99 | 71.69 |
| RFPBV12 | ON907881 | feces | Feces02 | 1614 | AVX53292 | Marmot picobirnavirus | 99 | 75.88 |
| RFPBV13 | ON907882 | feces | Feces02 | 1458 | QXV86690 | Picobirnavirus sp. | 98 | 63.12 |
| RFPBV14 | ON907883 | feces | Feces03 | 1191 | AVX53270 | Marmot picobirnavirus | 99 | 81.54 |
| RFPBV15 | ON907884 | feces | Feces03 | 1623 | QXV86679 | Picobirnavirus sp. | 99 | 70.90 |
| 1RF1-MCP | ON840016 | feces | Feces01 | 5149 | AYQ58162 | Microviriade sp. | 99 | 99.97 |
| 1RF2-MCP | ON840017 | feces | Feces01 | 3359 | AYQ58162 | Microviriade sp. | 99 | 58.26 |
| 1RF3-MCP | ON840018 | feces | Feces01 | 4856 | QRV61993 | Microviriade sp. | 33 | 45.45 |
| 1RF4-MCP | ON840019 | feces | Feces01 | 3640 | DAF98539 | Microviriade sp. | 99 | 88.24 |
| 1RF5-MCP | ON840020 | feces | Feces01 | 5255 | DAV98530 | Microviriade sp. | 31 | 72.63 |
| 1RF6-MCP | ON840021 | feces | Feces01 | 5068 | QJB20933 | Microviriade sp. | 36 | 32.72 |
| 1RO1-MCP | ON840022 | oralswab | Oralswab01 | 5500 | UCS96105 | Microviriade sp. | 99 | 57.29 |
| 1RS1-MCP | ON840023 | skinswab | Skinswab01 | 5716 | QPB07478 | Microvirus sp. | 28 | 62.79 |
| 1RS2-MCP | ON840024 | skinswab | Skinswab01 | 4254 | QJB20795 | Microvirus sp. | 29 | 67.85 |
| 1RS3-MCP | ON840025 | skinswab | Skinswab01 | 3154 | QCS36979 | Tortoise microvirus 38 | 99 | 69.96 |
| 1RS4-MCP | ON840026 | skinswab | Skinswab01 | 4934 | QJB20344 | Microvirus sp. | 33 | 37.01 |
| 2RF1-MCP | ON840027 | feces | Feces02 | 4562 | QXP45030 | Microvirus mar8 | 37 | 47.49 |
| 2RF2-MCP | ON840028 | feces | Feces02 | 6397 | DAD87929 | Microvirus sp.ctfjj5 | 99 | 86.27 |
| 2RF3-MCP | ON840029 | feces | Feces02 | 5230 | DAX03554 | Microviriade sp. | 27 | 95.81 |
| 2RF4-MCP | ON840030 | feces | Feces02 | 4889 | DAU01201 | Microviriade sp. | 99 | 49.46 |
| 2RF5-MCP | ON840031 | feces | Feces02 | 4256 | DAQ92968 | Microviriade sp. | 39 | 73.08 |
| 2RF6-MCP | ON840032 | feces | Feces02 | 2354 | QRV61993 | Microvirus sp. | 98 | 34.72 |
| 2RF7-MCP | ON840033 | feces | Feces02 | 4545 | DAS73481 | Microviriade sp. | 99 | 67.79 |
| 2RO1-MCP | ON840034 | oralswab | Oralswab02 | 3315 | QJB19341 | Microvirus sp. | 26 | 82.03 |
| 3RF1-MCP | ON840035 | feces | Feces03 | 4716 | DAU01201 | Microviriade sp. | 37 | 49.56 |
| 3RF2-MCP | ON840036 | feces | Feces03 | 5447 | QXN75191 | Microvirus sp.mar39 | 33 | 66.55 |
| 3RF3-MCP | ON840037 | feces | Feces03 | 3834 | DAS73481 | Microviriade sp. | 99 | 60.79 |
| 3RF4-MCP | ON840038 | feces | Feces03 | 4917 | QPB07363 | Microvirus sp. | 34 | 53.05 |
| 3RF5-MCP | ON840039 | feces | Feces03 | 5087 | DAR43455 | Microviriade sp. | 33 | 71.29 |
| 3RF6-MCP | ON840040 | feces | Feces03 | 5344 | DAN06693 | Microviriade sp. | 30 | 73.02 |
| 3RF7-MCP | ON840041 | feces | Feces03 | 2680 | DAV68623 | Microviriade sp. | 61 | 46.38 |
| 4RF1-MCP | ON840042 | feces | Feces04 | 4778 | QPB07359 | Microvirus sp. | 34 | 58.71 |
| 4RF2-MCP | ON840043 | feces | Feces04 | 6106 | DAJ78944 | Microvirus sp. | 29 | 92.01 |
| 4RF3-MCP | ON840044 | feces | Feces04 | 4680 | DAJ87275 | Microviriade sp. | 43 | 66.67 |
| 4RF4-MCP | ON840045 | feces | Feces04 | 6110 | DAH93073 | Microviriade sp. | 73 | 88.76 |
| 4RF5-MCP | ON840046 | feces | Feces04 | 5403 | DAR43455 | Microvirus mar24 | 30 | 70.32 |
| 4RF6-MCP | ON840047 | feces | Feces04 | 2611 | QXN75098 | Microvirus mar12 | 99 | 63.35 |
| 4RF7-MCP | ON840048 | feces | Feces04 | 3339 | QXN75036 | Microviriade sp. | 90 | 50.43 |
| 1RF1-TERL | ON840049 | feces | Feces01 | 1875 | DAR43123 | Siphoviriade sp. | 39 | 98.24 |
| 1RF2-TERL | ON840050 | feces | Feces01 | 1776 | WP_163588178 | Ligilactobacillus murinus | 34 | 99.49 |
| 1RF3-TERL | ON840051 | feces | Feces01 | 1554 | DAD92946 | Siphoviriade sp. | 80 | 98.06 |
| 1RO1-TERL | ON840052 | oralswab | Oralswab01 | 1647 | YP_009149970 | Leuconostoc phage Ln-9 | 99 | 68.67 |
| 1RO2-TERL | ON840053 | oralswab | Oralswab01 | 1623 | YP_009877815 | Leuconostoc phage 05601 | 99 | 97.04 |
| 1RO3-TERL | ON840054 | oralswab | Oralswab01 | 1614 | DAV26782 | Siphoviriade sp. | 99 | 94.23 |
| 1RS1-TERL | ON840055 | skinswab | Skinswab01 | 1566 | QXN17376 | Rhodobacter phage RcHartney | 97 | 59.31 |
| 1RS2-TERL | ON840056 | skinswab | Skinswab01 | 1224 | YP_009191426 | Enterobacter phage phiEap-2 | 99 | 97.31 |
| 1RS3-TERL | ON840057 | skinswab | Skinswab01 | 1746 | QJD49730 | Streptomyces phage Clubpeguin | 99 | 72.98 |
| 1RS4-TERL | ON840058 | skinswab | Skinswab01 | 1752 | QAY17126 | Streptomyces phage Madamato | 99 | 80.96 |
| 2RF1-TERL | ON840059 | feces | Feces02 | 1509 | DAU91043 | Myoviriade sp. | 99 | 99.81 |
| 2RF2-TERL | ON840060 | feces | Feces02 | 1293 | DAP09869 | Siphoviriade sp. | 95 | 64.34 |
| 2RF3-TERL | ON840061 | feces | Feces02 | 1497 | DAP09869 | Siphoviriade sp. | 80 | 63.79 |
| 2RO1-TERL | ON840062 | oralswab | Oralswab02 | 1149 | DAZ21572 | Siphoviriade sp. | 88 | 62.21 |
| 2RO2-TERL | ON840063 | oralswab | Oralswab02 | 1785 | DAZ21573 | Siphoviriade sp. | 85 | 69.01 |
| 2RO3-TERL | ON840064 | oralswab | Oralswab02 | 1755 | DAY14861 | Siphoviriade sp. | 90 | 69.35 |
| 2RO4-TERL | ON840065 | oralswab | Oralswab02 | 1746 | DAY14861 | Siphoviriade sp. | 92 | 69.77 |
| 2RS1-TERL | ON840066 | skinswab | Skinswab02 | 1149 | DAZ21572 | Siphoviriade sp. | 99 | 61.82 |
| 2RS2-TERL | ON840067 | skinswab | Skinswab02 | 1743 | DAN58510 | Siphoviriade sp. | 100 | 77.27 |
| 2RS3-TERL | ON840068 | skinswab | Skinswab02 | 1746 | DAN58510 | Siphoviriade sp. | 98 | 78.21 |
| 3RF2-TERL | ON840069 | feces | Feces03 | 1125 | UCS96242 | Siphoviriade sp. | 99 | 66.41 |
| 3R01-TERL | ON840070 | oralswab | Oralswab03 | 1653 | DAD97146 | Siphoviriade sp. | 99 | 76.01 |
| 3R02-TERL | ON840071 | oralswab | Oralswab03 | 1302 | ALY10643 | Arthrobacter phage Taj14 | 97 | 49.42 |
| 3R03-TERL | ON840072 | oralswab | Oralswab03 | 1467 | DAZ21572 | Siphoviriade sp. | 92 | 64.01 |
| 4RF1-TERL | ON840073 | feces | Feces04 | 1167 | DAE40686 | Siphoviriade sp. | 95 | 62.23 |
| 4RF2-TERL | ON840074 | feces | Feces04 | 1053 | DAP09869 | Siphoviriade sp. | 92 | 42.31 |
| 1RF1-PodoTERL | ON840075 | feces | Feces01 | 1542 | YP_006906086 | Escherichia phage NJ01 | 99 | 99.81 |
| 1RO1-PodoTERL | ON840076 | oralswab | Oralswab01 | 1467 | DAI36110 | Podoviriade sp. | 97 | 71.34 |
| 1RO2-PodoTERL | ON840077 | oralswab | Oralswab01 | 1602 | DAS51038 | Podoviriade sp. | 99 | 74.42 |
| 3RO1-PodoTERL | ON840078 | oralswab | Oralswab03 | 1485 | AVJ48276 | Salmonella phage SE131 | 99 | 92.71 |
| 1RF2-MyoTERL | ON840079 | feces | Feces01 | 1254 | DAK19831 | Myoviriade sp. | 26 | 74.31 |
| 1RS1-MyoTERL | ON840080 | skinswab | Skinswab01 | 1801 | QVW53830 | Acinetobacter phage TaPaz | 98 | 54.99 |
| 1RO1-AutographTERL | ON840081 | oralswab | Oralswab01 | 1731 | YP_009005154 | Cronobacter phage Dev2 | 99 | 98.61 |
